# 17β-Estradiol and Its Metabolites Induce Oxidative Damage to Membrane Lipids in Primary Porcine Thyroid Follicular Cells—Comparison Between Sexes

**DOI:** 10.3390/ijms262411807

**Published:** 2025-12-06

**Authors:** Jan Stępniak, Małgorzata Karbownik-Lewińska

**Affiliations:** 1 Department of Endocrinology and Metabolic Diseases, Medical University of Lodz, 281/289 Rzgowska St., 93-338 Lodz, Poland; jan.stepniak@umed.lodz.pl; 2 Polish Mother’s Memorial Hospital—Research Institute, 281/289 Rzgowska St., 93-338 Lodz, Poland

**Keywords:** thyroid, 17β-estradiol, estradiol metabolites, lipid peroxidation, oxidative damage, sexual dimorphism

## Abstract

Sexual dimorphism significantly influences the epidemiology of thyroid disorders, with females exhibiting higher incidence of thyroid diseases. Estrogens and their hydroxylated metabolites are key regulators of cellular redox balance and may contribute to sex-specific susceptibility through pro-oxidative mechanisms. However, the impact of individual estrogen metabolites on oxidative stress in thyroid follicular cells remains poorly defined. Here, we investigated the pro-oxidative effects of 17β-estradiol (E2) and its hydroxylated metabolites—2-hydroxyestradiol (2-OHE2), 4-hydroxyestradiol (4-OHE2), and 16α-hydroxyestrone (16α-OHE1)—in primary porcine thyroid cell cultures from males and females. Primary follicular thyroid cells were isolated from six male and six female pigs. Cells were exposed to E2 (100 nM) or its metabolites (1 μM), with or without Fenton reaction substrates (Fe^2+^ and H_2_O_2_), for 24 h. Lipid peroxidation (an index of oxidative damage to lipids) was quantified using BODIPY^®^ 581/591 C11 fluorescence via flow cytometry. Basal lipid peroxidation did not differ between sexes. 2-OHE2 increased lipid peroxidation in both male and female thyroid cells, with a more pronounced effect observed in males. In contrast, 4-OHE2 selectively enhanced lipid peroxidation only in female cells. 16α-OHE1 elevated lipid peroxidation in both sexes. E2 significantly increased lipid peroxidation in both male and female cells. Among all compounds tested, E2 exhibited the most potent pro-oxidative activity, particularly in female-derived cells. These findings provide novel insights into the redox-modulating effects of estrogen metabolism in the thyroid and suggest a potential molecular basis for sex-related susceptibility to thyroid dysfunction. While based on an in vitro porcine model, the study increases our understanding of the mechanisms by which estrogenic compounds may influence thyroid pathophysiology, possibly including early events in thyroid disease development or oncogenesis.

## 1. Introduction

Sexual dimorphism is a recognized factor in the epidemiology of many human diseases. This sex-based disparity is particularly pronounced in thyroid disorders, where females exhibit a three- to four-fold higher incidence of both benign and malignant neoplasms and a greater susceptibility to autoimmune conditions compared to males [1,2]. The mechanisms driving this female predisposition are not fully elucidated, but evidence points towards the differential metabolism of sex hormones as a key contributing factor [3,4]. The influence of estrogen metabolism on disease is not limited to the thyroid, as sex-specific patterns in cardiovascular diseases [5], airway diseases [6], and other conditions [7] are also linked to the local production and action of estrogen metabolites. This suggests that sex-dependent variations in estrogen processing may be a fundamental mechanism underlying disease vulnerability.

Estrogens, primarily 17β-estradiol (E2) and its precursor estrone (E1), are essential for reproductive function but also regulate proliferation, differentiation, and apoptosis in multiple non-reproductive tissues [6]. Their biological activity is further shaped by metabolic conversion into distinct metabolites with sometimes opposing effects. In Phase I, cytochrome P450 enzymes hydroxylate E2/E1 at the C-2 and C-4 positions, yielding 2-hydroxyestrogens (2-OHE) and 4-hydroxyestrogens (4-OHE), respectively [8]. These catechol estrogens can either undergo Phase II conjugation by enzymes such as catechol-O-methyltransferase (COMT) and UDP-glucuronosyltransferases (UGTs), generating stable and readily excreted metabolites, or be oxidized to quinone/semiquinone species that form DNA adducts and promote redox cycling with excess reactive oxygen species (ROS) generation [9,10]. An increased ratio of 4-OHE to 2-OHE is associated with a shift towards this “genotoxic” pathway and a higher risk of hormone-dependent cancers [11].

Individual estrogen metabolites exhibit distinct biochemical behaviors that may influence disease risk. 2-Hydroxyestradiol (2-OHE2), primarily formed by CYP1A1 in the liver [12], is weakly estrogenic and can generate ROS at high concentrations [13] but also shows antioxidant properties by protecting against oxidative stress [14]. 4-Hydroxyestradiol (4-OHE2), mainly produced by CYP1B1 in extrahepatic tissues, has strong estrogenic activity and is widely considered pro-oxidant due to its quinone redox cycling and DNA-damaging potential, though it can also exert antioxidant effects under certain conditions [12,15]. 16α-Hydroxyestrone (16α-OHE1), formed primarily via CYP3A4-mediated hydroxylation, is highly estrogenic and promotes cell proliferation, with a debated pro-oxidant role but recognized ability to enhance detoxification through stimulation of glutathione S-transferase (GST) activity [8,12].

Our previous research has demonstrated a clear link between E2 and oxidative processes within the thyroid gland. In a study using primary thyroid cell cultures derived from Wistar rats, we showed that the expression of the H_2_O_2_-generating enzymes, i.e., Dual oxidase 1 (DUOX1) and NADPH oxidase 4 (NOX4) were significantly higher in cells from females compared to males [16]. This finding is consistent with other experimental evidence indicating sexual dimorphism in NOX4 expression [17] and is particularly relevant given that NOX4 is frequently overexpressed in thyroid cancer [18]. Additionally, we found that estrogen excess may contribute to the upregulation of key thyroid-specific genes such as thyroperoxidase (TPO), the activation of endoplasmic reticulum stress pathways via genes like C/EBP homologous protein (CHOP), and the direct stimulation of the NOX/DUOX system, ultimately leading to increased reactive oxygen species (ROS) production [19].

As thyroid tissue exhibits intrinsic sexual dimorphism in its oxidative state, with female thyroid cells displaying higher basal levels of H_2_O_2_ due to greater expression of DUOX/NOX enzymes [16,17], it can be hypothesized that the increased female susceptibility to thyroid disease results from the convergence of this elevated basal oxidative stress with the genotoxic potential of local estrogen metabolism. Specifically, an imbalance favoring the production of 4-OHE and its subsequent oxidation to quinones may amplify oxidative damage in the already-stressed female thyroid.

Despite growing recognition of the role of estrogens in redox regulation and our prior findings that E2 may exert both protective and pro-oxidative effects depending on tissue type, dose, and sex, the specific contribution of individual estrogen metabolites to oxidative processes in the thyroid remains poorly understood. While previous studies in thyroid homogenates demonstrated that E2 reduced Fenton reaction-induced lipid peroxidation [20,21,22], our current research moves beyond homogenate models to examine the direct effects of E2 and its major metabolites (2-OHE2, 4-OHE2, and 16α-OHE1) in a more physiologically relevant model using primary porcine thyroid cell cultures derived from both sexes. Importantly, we employed a fluorescent lipid peroxidation probe (BODIPY^®^ 581/591 C11) that selectively localizes to cellular membranes in live cells, allowing real-time, compartment-specific detection of oxidative damage. This contrasts with previous spectrophotometric measurements in tissue homogenates, where lipid peroxidation was assessed globally across mixed cellular compartments, potentially masking subcellular specificity. This approach allows us to investigate the sex-specific oxidative effects of E2 and its major metabolites under both basal and pro-oxidant (Fenton reaction) conditions, offering new insights into estrogen metabolism and its potential role in thyroid disease vulnerability.

This study therefore investigates the relationship between sex-specific impact of estrogen metabolites and oxidative damage in thyroid cells to elucidate a potential hormonal mechanism for the sexual dimorphism observed in thyroid disease.

**Figure 1:**
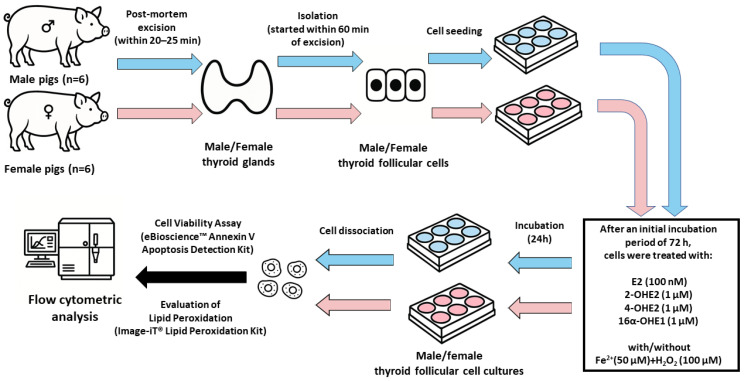
Study design diagram.

The study design diagram is presented in Figure 1.

## 2. Results

To verify that the applied concentrations of E2 and its metabolites were not cytotoxic, cell viability was assessed by Annexin V/propidium iodide (PI) staining. Across all tested conditions, including treatments in the presence and absence of Fenton reaction substrates, no significant changes in the proportions of viable, early apoptotic, or late apoptotic/necrotic cells were observed compared with vehicle-treated controls (Supplementary Figure S1). These findings indicate that the concentrations used in this study did not induce overt cell death and that the observed changes in lipid peroxidation reflect oxidative stress in predominantly viable thyroid cell populations.

**Figure 2:**
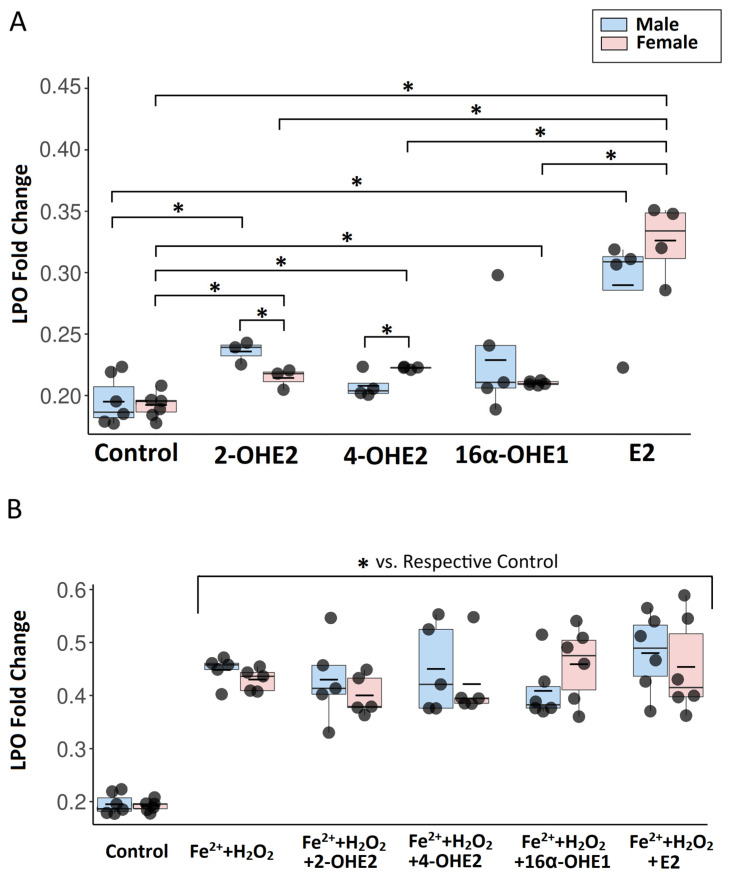
The graph presents a comparison of the effects of individual estradiol metabolites (2-OHE2, 4-OHE2, 16α-OHE1) and E2 on lipid peroxidation in female- and male-derived thyroid cells in basal conditions (**A**) and with Fenton reaction substrates (**B**). Lipid peroxidation in cells was quantified based on the ratio of fluorescence signals at 590 nm (red—before oxidation) to 510 nm (green—after oxidation). To enhance data visualization, results are expressed as 1/(590/510 ratio). The central line represents the median, the box spans the interquartile range (25th to 75th percentile), and the whiskers indicate the minimum and maximum values, the short horizontal line denotes the mean. *n* ≥ 3 independent experiments. * *p* < 0.05.

Flow cytometry analysis revealed no significant differences in basal lipid peroxidation between male and female porcine thyroid cells. To assess the effect of 2-OHE2 on lipid peroxidation, a concentration of 1 μM was applied to primary thyroid cell cultures derived from male and female pigs for 24 h. Flow cytometry analysis revealed that 2-OHE2 increased lipid peroxidation in both male and female porcine thyroid cells, with a more pronounced pro-oxidative effect observed in male cells (Figure 2A). After incubation with both Fenton reaction substrates and 2-OHE2, lipid peroxidation levels were significantly elevated compared to control cells and cells treated with 2-OHE2 alone; however, no significant differences were observed relative to cells exposed only to Fenton reaction substrates (Figure 2B).

Incubation of porcine thyroid cells with 1 μM of 4-OHE2 for 24 h led to a significant increase in lipid peroxidation only in female-derived cells, whereas no significant change was observed in male cells (Figure 2A). After incubation with both Fenton reaction substrates and 4-OHE2, lipid peroxidation levels were significantly elevated compared to control cells and those treated with 4-OHE2 alone; however, no significant differences were observed relative to cells exposed only to Fenton reaction substrates (Figure 2B).

Incubation of porcine thyroid cells with 1 μM of 16α-OHE1 for 24 h led to a significant increase in lipid peroxidation in both male- and female-derived cells (Figure 2A). After incubation with both Fenton reaction substrates and 16α-OHE1, lipid peroxidation levels were significantly elevated compared to control cells and to those treated with 16α-OHE1 alone; however, no significant differences were observed relative to cells exposed only to Fenton reaction substrates (Figure 2B).

Exposure of porcine thyroid cells to 1 μM E2 for 24 h resulted in a significant increase in lipid peroxidation in both male- and female-derived cells (Figure 2A). After incubation with both Fenton reaction substrates and E2, lipid peroxidation levels were significantly elevated compared to control cells and those treated with E2 alone; however, no significant differences were observed relative to cells exposed only to Fenton reaction substrates (Figure 2B).

Exposure of porcine thyroid cells to 1 μM E2 and its metabolites (2-OHE2, 4-OHE2, and 16α-OHE1) for 24 h revealed sex-dependent differences in lipid peroxidation. In female-derived cells, E2 induced the marked increase in lipid peroxidation, with levels significantly higher than those observed after treatment with 2-OHE2, 4-OHE2, or 16α-OHE1. In male-derived cells, the effect of E2 was significantly greater only when compared with 4-OHE2, while no significant differences were found relative to 2-OHE2 or 16α-OHE1. Additionally, in male cells, lipid peroxidation induced by 2-OHE2 was significantly higher than that observed after 4-OHE2 treatment. No other significant differences between metabolites were detected. These findings indicate that E2 exerts the most pronounced pro-oxidative effect, particularly in female thyroid cells, highlighting potential sex-related differences in susceptibility to estrogen-induced oxidative stress (Figure 2A).

## 3. Discussion

Understanding sex differences is critical for advancing biomedical knowledge, as these variations influence disease susceptibility, pathological processes, and therapeutic outcomes. Historically, research on sexual dimorphism has concentrated on reproductive tissues, including the gonads, external genitalia, and brain regions involved in reproductive behaviors, where anatomical and functional differences between sexes are most evident. However, recent studies have demonstrated that sex differences extend across nearly all physiological systems, significantly affecting metabolism, immune function, and cardiovascular health, with important clinical implications [2]. A key contributor to many of these non-reproductive sex differences may be the metabolism of sex hormones, particularly estrogens. It has been shown that not only the parent sex hormones but also their metabolites can exert distinct or even opposing physiological effects [8,13,14,15]. Moreover, the balance between protective metabolic pathways, such as 2-hydroxylation, and potentially genotoxic pathways, like 4-hydroxylation, creates a sex-specific biochemical milieu that influences tissue health and disease risk.

Our results provide insights into the sex-specific pro-oxidative effects of estrogen metabolites in porcine thyroid cells, supporting and extending previous findings from other tissues and model systems. Importantly, we found that basal lipid peroxidation did not differ between male and female porcine thyroid cells, suggesting that any sex-specific differences observed following treatment were not attributable to intrinsic disparities in basal oxidative state. This observation is consistent with our earlier findings in porcine thyroid cultures, where no sex-related differences were detected in basal levels of intracellular ROS, as measured by flow cytometry using CellROX™ Orange Reagent (ThermoFisher, Waltham, MA, USA) [19]. However, it should be noted that in a previous study using primary thyroid cell cultures derived from Wistar rats, we found that female thyroid cells were intrinsically exposed to higher concentrations of H_2_O_2_, likely due to elevated expression of DUOX/NOX enzymes [16]. Together, these findings suggest that species-specific or compartmentalized redox mechanisms may underlie the observed responses.

Exposure to 2-OHE2 led to a significant increase in lipid peroxidation in both male- and female-derived thyroid cells, with a notably stronger effect observed in the male group. This finding is particularly noteworthy given previous reports describing the dual role of 2-OHE2, which can function as both an antioxidant and a pro-oxidant depending on its concentration and cellular context. While 2-OHE2 has been shown to reduce oxidative stress and protect against the accumulation of ROS and lipid peroxidation in hepatoma cells [23], as well as prevent H_2_O_2_-induced cell death in neuronal cells [14], it has also been reported to induce ROS accumulation, 8-oxo-7,8-dihydro-2′-deoxyguanosine formation, cytotoxicity, and disruption of mitochondrial membrane potential in cultured human mammary epithelial cells, likely through ROS-mediated mechanisms [13]. Our results suggest that, in porcine thyroid cells, the pro-oxidant activity of 2-OHE2 predominates—particularly in male cells—which may be attributed to sex-specific differences in redox enzyme expression or quinone detoxification capacity.

In contrast, 4-OHE2 significantly increased lipid peroxidation only in female-derived thyroid cells, with no observable effect in male cells. This metabolite is widely recognized as a potent pro-oxidant and tumor initiator, capable of undergoing redox cycling and forming DNA-damaging quinones. Notably, 4-OHE2 is considered to have greater carcinogenic potential than 2-OHE2. However, this enhanced potency is not solely attributable to the generation of hydroxyl radicals via redox cycling, as both metabolites exhibit comparable redox potentials. Instead, it is likely due to the substantially higher levels of depurinating DNA adducts formed by catechol quinones derived from 4-OHE2 compared to those generated from 2-OHE2 [24]. Although some studies have also highlighted the cytoprotective potential of 4-OHE2 under specific stress conditions, such as its ability to reduce chromium VI-induced lipid peroxidation in mitochondria and erythrocytes and to increase superoxide dismutase (SOD) activity, these effects suggest a role in mitigating oxidative stress [25]. Interestingly, these protective effects are not primarily attributed to simple chemical antioxidant properties, as the reductive capacity of the phenolic moiety does not appear to be the main mechanism underlying the observed actions [26]. Our findings are consistent with the genotoxic and pro-oxidative profile of 4-OHE2, particularly in female thyroid tissue. The observed sex-specific susceptibility may reflect higher basal expression of estrogen receptors (ERs) or differences in metabolic and detoxification pathway activity in female thyroid cells.

Similarly, 16α-OHE1 induced a significant increase in lipid peroxidation; however, unlike 4-OHE2, this effect was observed in both male- and female-derived thyroid cells. This metabolite is known for its strong estrogenic and proliferative activity, and although its direct pro-oxidant role remains debated, several studies have demonstrated its genotoxic potential through covalent binding to macromolecules, including DNA and proteins [27]. Furthermore, 16α-OHE1 has been shown to enhance the production of superoxide anion and hydrogen peroxide by activating ERα, leading to upregulation of NADPH oxidase 1 and 4 [28]. Our findings are consistent with these observations, providing evidence that 16α-OHE1 exerts pro-oxidant effects in thyroid cells of both sexes. This broader effect compared to 4-OHE2 may indicate a mechanism less dependent on sex-specific metabolic or detoxification pathways and more closely tied to its potent estrogenic activity and ability to activate redox enzymes.

Increased lipid peroxidation following E2 exposure is consistent with our previous demonstration that E2 induces dose-dependent ROS formation in thyroid cells [19]. Here, 1 μM E2 for 24 h significantly increased lipid peroxidation in both male- and female-derived cells, and this effect was further enhanced in the presence of Fenton substrates. Notably, E2 showed the strongest pro-oxidative potential among the compounds tested, particularly in female cells, exceeding the effects of 2-OHE2, 4-OHE2, and 16α-OHE1. This suggests that the parent hormone may exert a more direct and potent influence on redox balance than its metabolites, possibly reflecting its higher affinity for estrogen receptors and greater metabolic stability [29,30,31,32]. Together with the sex-specific responses observed, these findings support the hypothesis that female thyroid tissue is inherently more vulnerable to estrogen-induced oxidative damage due to a combination of receptor-mediated signaling, metabolite redox cycling, and differences in detoxification pathways.

The observed sex-specific responses may reflect underlying differences in estrogen receptor expression and activity between male and female thyroid tissue. Studies in rodents indicate that both ERα and ERβ are expressed in thyroid cells of both sexes, but females exhibit higher overall receptor levels, likely due to higher circulating E2 concentrations [33]. Moreover, ERβ expression peaks transiently in male thyroids during postnatal development, coinciding with increased proliferation and TSH receptor expression, suggesting complex temporal and sex-dependent regulation. Additionally, cytochrome P450 enzymes involved in estrogen metabolism, such as CYP1A1 and CYP1B1, are expressed in the thyroid and show higher expression in females in other tissues, although sex-specific data for thyroid remain lacking [34]. These differences in receptor levels and metabolic enzyme activity could contribute to the sex-dependent oxidative effects observed in our study. It is also plausible that variations in Phase II detoxification pathways, including COMT-mediated O-methylation and glutathione-dependent conjugation of estrogen quinones, as well as differences in NOX/DUOX-derived ROS generation and antioxidant defenses, further modulate the balance between pro- and antioxidative actions of E2 and its metabolites in a sex-dependent manner.

This study has several limitations that should be considered when interpreting the results. The experiments were conducted using in vitro porcine thyroid cell cultures, which, although physiologically relevant, cannot fully replicate the complexity of in vivo thyroid tissue, including systemic hormonal regulation, immune responses, and metabolic interactions. The study also utilized a single concentration of E2 and each estrogen metabolite, whereas dose–response analyses could provide a more comprehensive understanding of their redox behavior, potential biphasic or threshold effects, and sex-dependent sensitivity; future studies will therefore include extended dose–response and time-course experiments. The experiments focused exclusively on short-term exposure (24 h), leaving the potential long-term consequences of repeated or chronic exposure unexplored. We did not perform formal a priori power calculations, which limits our ability to exclude small effect sizes with high confidence; however, the observed variability and effect sizes are in line with our previous work and support the robustness of the main findings. We also did not perform additional functional assays (e.g., mitochondrial function or long-term viability/proliferation), which limits the direct linkage between lipid peroxidation and downstream cellular consequences, nor did we conduct parallel molecular assays (e.g., qPCR or Western blotting), which constrains direct mechanistic attribution of the observed sex differences to specific receptor and metabolic pathways. Future studies will address these limitations by integrating detailed dose–response and time-course designs with mitochondrial, viability, and molecular profiling assays to more comprehensively characterize sex-dependent estrogen actions in thyroid cells.

Our findings, which demonstrate pro-oxidative effects of E2 in live thyroid cells, contrast with our earlier studies in porcine thyroid homogenates, where E2 exhibited predominantly antioxidative properties [20,21,22]. In homogenates, E2 attenuated Fenton-induced lipid peroxidation, particularly in male tissues [20], and protected membrane lipids and nuclear DNA in ovary and liver models [22,35]. These data point to the dual redox nature of E2, which appears highly dependent on biological context. In homogenates, E2 likely acts mainly as a chemical antioxidant, whereas in live cells it engages receptor-mediated signaling, mitochondrial pathways, and conversion to reactive metabolites, shifting the balance towards pro-oxidative outcomes. This underscores the importance of considering cellular metabolism and compartmentalization when interpreting estrogen redox actions.

In conclusion, E2 and its hydroxylated metabolites exert distinct and sex-dependent pro-oxidative effects in porcine thyroid cells. Among the compounds tested, E2 showed the strongest ability to induce lipid peroxidation under tested conditions, while 2-OHE2 exhibited greater pro-oxidant activity in males, 4-OHE2 selectively affected females, and 16α-OHE1 increased lipid peroxidation in both sexes. These findings suggest that estrogen metabolites, beyond their classical receptor-mediated roles, may contribute to oxidative stress in thyroid cells in a sex-dependent manner. While not directly translatable to human pathology, this observation could help inform hypotheses about the biological basis of sex differences in thyroid disorders. Future investigations should include dose–response analyses, long-term exposures, and in vivo validation to better define the translational relevance of these observations for human thyroid pathophysiology.

## 4. Materials and Methods

Most reagents (including E2, purity ≥ 98%; Item No. E1024) were purchased from Sigma-Aldrich (St. Louis, MO, USA). 2-OHE2 (purity ≥ 95%; Item No. 34154), 4-OHE2 (purity ≥ 95%; Item No. 31508) and 16α-OHE1 (purity ≥ 98%; Item No. 15208) were purchased from Cayman Chemical Company (Ann Arbor, MI, USA). Other cases are specified.

### 4.1. Animals

Thyroid glands were obtained from six (6) adult male and six (6) adult female pigs, approximately 8–9 months old, sourced from a commercial slaughterhouse. All procedures related to animal slaughter conformed to European Community Council Regulation (EC) No. 1099/2009. It is important to note that no approval from a Local Ethics Committee is required for the collection of tissues or organs from animals slaughtered for commercial purposes, provided the collection site is registered in accordance with Directive 2010/63/EU of the European Parliament and of the Council on the protection of animals used for scientific purposes (published 22 September 2010), as well as the Polish Act on the Protection of Animals Used for Scientific or Educational Purposes (published 15 January 2015).

The animals were approximately 8–9 months old, indicating sexual maturity, with a mean body weight of 115.17 ± 8.7 kg (SD). Sex was initially assessed by a veterinarian using primary external sexual characteristics, specifically the position of the urogenital opening, and subsequently verified during thoracic opening and evisceration by confirming the presence or absence of reproductive organs such as the uterus and ovaries. The attending veterinarian certified that all animals were healthy and free of visible pathological lesions. Thyroid glands were excised post-mortem within 20–25 min after slaughter, transferred to Hanks’ balanced salt solution (HBSS), transported on ice, and processed for cell isolation within 60 min of excision.

### 4.2. Cell Culture

Primary thyroid follicular cells from each animal were isolated and seeded into multiple culture wells so that each experimental condition was represented once per donor. For the present study, only cultures that met predefined morphological and viability criteria were included in the analyses; therefore, the final number of independent experiments (*n*) may differ slightly between specific measurements.

Any residual connective tissue was carefully removed from the thyroid samples. The tissue was finely chopped using a sterile razor blade to produce fragments approximately 1–3 mm in size. These fragments were rinsed twice with HBSS and subsequently subjected to enzymatic digestion in collagenase IV solution (2 mg/mL; Item No. C5138) for approx. 1.5 to 3 h at 37 °C. The resulting cell suspension was passed through a 100 μm nylon mesh to eliminate undigested material. Following two additional washes with HBSS and centrifugation at 1000× *g* for 5 min, the isolated cells were seeded at a density of 1 × 10^6^ cells/mL into 6-well culture plates containing 1 mL of medium per well.

Phase-contrast microscopy, together with collagenase-based isolation and filtration, indicated that the primary cultures contained >95% thyroid follicular cells, with only a minor stromal component.

The culture medium used was DMEM + GlutaMAX (without pyruvate; Gibco, Grand Island, NY, USA; Item No. 10566016), supplemented with 10% fetal bovine serum (FBS, Gibco, Grand Island, NY, USA), thyroid-stimulating hormone (TSH; 1 mIU/mL), penicillin-streptomycin (100 IU/mL), and amphotericin B (2.5 μg/mL). After an initial incubation period of 72 h at 37 °C in a humidified atmosphere of 95% air and 5% CO_2_, the test compounds were introduced to the cultures.

### 4.3. Cell Treatment

The cells of male or female origin were treated with E2 (100 nM), 2-OHE2 (1 μM), 4-OHE2 (1 μM) or 16α-OHE1 (1 μM) with/without Fenton reaction substrates, i.e., Fe^2+^ (50 μM) and H_2_O_2_ (100 μM). The selected concentrations and 24 h exposure time were based on our previous experiments in thyroid and other endocrine tissues, where these conditions elicited reproducible effects without inducing cytotoxicity [16,19,22]. Stock solutions of E2 and its metabolites were prepared in dimethyl sulfoxide (DMSO). Thus, control cells were also treated with DMSO (in the final concentration of 0.1%). The cells were incubated for another 24 h in the presence of examined substances.

### 4.4. Cell Viability Assay

A cell viability test was conducted using the eBioscience™ Annexin V Apoptosis Detection Kit (Invitrogen, Waltham, MA, USA; Item No. 88-8005-74) according to the manufacturer’s instructions and analyzed via flow cytometry with a FACSCanto II cytometer (BD FACSCanto II, San Diego, CA, USA). The data were processed using FACSDiva software 6.1.2 (BD, San Diego, CA, USA).

### 4.5. Evaluation of Lipid Peroxidation Level

The level of lipid peroxidation was measured with the use of Image-iT^®^ Lipid Peroxidation Kit for live cell analysis (ThermoFisher, Waltham, MA, USA; Item No. C10445) according to the manufacturer’s protocols. This method is based on BODIPY^®^ 581/591 C11 (ThermoFisher, Waltham, MA, USA) reagent and is a sensitive fluorescent reporter for lipid peroxidation. Upon oxidation in live cells, fluorescence shifts from red to green of the phenylbutadiene segment of the fluorophore 5, providing a ratiometric indication of lipid peroxidation by flow cytometry. Briefly, cells were treated with BODIPY^®^ 581/591 C11 reagent at a final concentration of 5 μM and incubated for 30 min at 37 °C. After incubation, cells were dissociated with the use of TrypLE™ (Gibco) enzyme and subjected to flow cytometry. Flow cytometry analysis was performed using the FACSCanto II cytometer (BD FACSCanto II, San Diego, CA, USA). Analysis of data was performed using the FACSDiva software (BD, San Diego, CA, USA).

### 4.6. Statistical Analysis

The number of independent experiments (*n*) was determined based on effect sizes observed in our previous studies using similar thyroid models and the practical constraints of primary porcine cell cultures. Therefore, a formal a priori sample size calculation was not performed.

We used either the unpaired *t*-test with Bonferroni correction for multiple comparisons or the one-way analysis of variance test followed by the Newman–Keuls multiple comparison test to determine statistically significant differences. The results are presented as mean ± S.E. of three independent experiments. Statistically significant differences are accepted at the level of *p* < 0.05.

## Data Availability

The original contributions presented in this study are included in the article/Supplementary Materials. Further inquiries can be directed to the corresponding author.

## Supplementary Materials

The following supporting information can be downloaded at https://www.mdpi.com/article/10.3390/ijms262411807/s1.

## Abbreviations

The following abbreviations are used in this manuscript:

E2	17β-estradiol
E1	Estrone
2-OHE	2-hydroxyestrogens
4-OHE	4-hydroxyestrogens
CYP	cytochrome P450
COMT	catechol-O-methyltransferase
UGTs	UDP-glucuronosyltransferases
4-OHE-Q	4-hydroxyestrogen quinone
ROS	reactive oxygen species
2-OHE2	2-Hydroxyestradiol
4-OHE2	4-Hydroxyestradiol
16α-OHE1	16α-Hydroxyestrone
DUOX1	Dual oxidase 1
NOX4	NADPH oxidase 4
SOD	superoxide dismutase

## References

[1] Nowak T.J., Muehlenbein M.P. (2025). Toward understanding sexual immune dimorphism in humans. Front. Immunol..

[2] McClements L., Kautzky-Willer A., Kararigas G., Ahmed S.B., Stallone J.N. (2025). The role of sex differences in cardiovascular, metabolic, and immune functions in health and disease: A review for “Sex Differences in Health Awareness Day”. Biol. Sex Differ..

[3] Zane M., Parello C., Pennelli G., Townsend D.M., Merigliano S., Boscaro M., Toniato A., Baggio G., Pelizzo M.R., Rubello D. (2017). Estrogen and thyroid cancer is a stem affair: A. preliminary study. Biomed. Pharmacother..

[4] Xie J., Wang J., Cui X. (2025). Research progress on estrogen and estrogen receptors in the occurrence and progression of autoimmune thyroid diseases. Autoimmun. Rev..

[5] Novella S., Gerdts E., Kararigas G. (2025). Divergent mechanisms of cardiovascular remodeling between men and women. Am. J. Physiol. Heart Circ. Physiol..

[6] Harvey B.J., McElvaney N.G. (2024). Sex differences in airway disease: Estrogen and airway surface liquid dynamics. Biol. Sex Differ..

[7] Shi Y., Ma J., Li S., Liu C., Liu Y., Chen J., Liu N., Liu S., Huang H. (2024). Sex difference in human diseases: Mechanistic insights and clinical implications. Signal. Transduct. Target. Ther..

[8] You Q., Song H., Zhu Z., Wang J., Wang R., Du M., Fu Y., Yuan J., Tan R. (2024). Decoding the enigmatic estrogen paradox in pulmonary hypertension: Delving into estrogen metabolites and metabolic enzymes. Cell. Mol. Biol. Lett..

[9] Cooke P.S., Nanjappa M.K., Ko C., Prins G.S., Hess R.A. (2017). Estrogens in Male Physiology. Physiol. Rev..

[10] Bolton J.L., Trush M.A., Penning T.M., Dryhurst G., Monks T.J. (2000). Role of quinones in toxicology. Chem. Res. Toxicol..

[11] Wen C.J., Wu L.X., Fu L.J., Shen D.Y., Zhang X., Zhang Y.W., Yu J., Zhou H.H. (2014). Preferential induction of CYP1A1 over CYP1B1 in human breast cancer MCF-7 cells after exposure to berberine. Asian Pac. J. Cancer Prev..

[12] Tsuchiya Y., Nakajima M., Yokoi T. (2005). Cytochrome P450-mediated metabolism of estrogens and its regulation in human. Cancer Lett..

[13] Hurh Y.J., Chen Z.H., Na H.K., Han S.Y., Surh Y.J. (2004). 2-Hydroxyestradiol induces oxidative DNA damage and apoptosis in human mammary epithelial cells. J. Toxicol. Environ. Health A.

[14] Zacharia L.C., Eleftheriou C., Gkretsi V. (2022). Effects of 2-methoxyestradiol on hydrogen peroxide induced neuronal cell death and tau hyperphosphorylation. Life Sci..

[15] Cavalieri E.L., Rogan E.G. (2004). A unifying mechanism in the initiation of cancer and other diseases by catechol quinones. Ann. N. Y. Acad. Sci..

[16] Stepniak J., Lewinski A., Karbownik-Lewinska M. (2018). Sexual Dimorphism of NADPH Oxidase/H_2_O_2_ System in Rat Thyroid Cells; Effect of Exogenous 17β-Estradiol. Int. J. Mol. Sci..

[17] Fortunato R.S., Braga W.M., Ortenzi V.H., Rodrigues D.C., Andrade B.M., Miranda-Alves L., Rondinelli E., Dupuy C., Ferreira A.C., Carvalho D.P. (2013). Sexual dimorphism of thyroid reactive oxygen species production due to higher NADPH oxidase 4 expression in female thyroid glands. Thyroid.

[18] Dang H., Sheng J., Tang P., Peng X., Zhang R., Zhao X., Hu J., Xu T. (2023). The role and mechanism of NADPH oxidase in the development and progression of thyroid carcinoma. Am. J. Cancer Res..

[19] Stępniak J., Karbownik-Lewińska M. (2024). 17β-Estradiol Stimulates Oxidative Stress Components and Thyroid Specific Genes in Porcine Thyroid Follicular Cells: Potential Differences Between Sexes. Cells.

[20] Stępniak J., Koziróg E., Karbownik-Lewińska M. (2023). The Protective Effect of Exogenous 17β-Estradiol against Experimentally Induced Oxidative Damage to Membrane Lipids Is Stronger in Male vs. Female Porcine Thyroids: Preliminary Results. Toxics.

[21] Rynkowska A., Stępniak J., Karbownik-Lewińska M. (2020). Fenton Reaction-Induced Oxidative Damage to Membrane Lipids and Protective Effects of 17β-Estradiol in Porcine Ovary and Thyroid Homogenates. Int. J. Environ. Res. Public Health.

[22] Stepniak J., Karbownik-Lewinska M. (2016). 17β-estradiol prevents experimentally-induced oxidative damage to membrane lipids and nuclear DNA in porcine ovary. Syst. Biol. Reprod. Med..

[23] Sun X., Hao X., Jia Y.C., Zhang Q., Zhu Y.Y., Yang Y.X., Zhu B.T. (2025). Protective effect of 2-hydroxyestrone and 2-hydroxyestradiol against chemically induced hepatotoxicity in vitro and in vivo. J. Pharmacol. Exp. Ther..

[24] Cavalieri E., Rogan E. (2006). Catechol quinones of estrogens in the initiation of breast, prostate, and other human cancers: Keynote lecture. Ann. N. Y. Acad. Sci..

[25] Sawicka E., Długosz A. (2017). The role of 17β-estradiol metabolites in chromium-induced oxidative stress. Adv. Clin. Exp. Med..

[26] Choi H.J., Lee A.J., Kang K.S., Song J.H., Zhu B.T. (2020). 4-Hydroxyestrone, an Endogenous Estrogen Metabolite, Can Strongly Protect Neuronal Cells Against Oxidative Damage. Sci. Rep..

[27] Stanczyk F.Z. (2024). Metabolism of endogenous and exogenous estrogens in women. J. Steroid Biochem. Mol. Biol..

[28] Hood K.Y., Montezano A.C., Harvey A.P., Nilsen M., MacLean M.R., Touyz R.M. (2016). Nicotinamide Adenine Dinucleotide Phosphate Oxidase-Mediated Redox Signaling and Vascular Remodeling by 16α-Hydroxyestrone in Human Pulmonary Artery Cells: Implications in Pulmonary Arterial Hypertension. Hypertension.

[29] Valencia C., Molina C., Florez M., Buñay J., Moreno R.D., Orihuela P.A., Castro A., Parada-Bustamante A. (2016). 2-hydroxyoestradiol and 2-methoxyoestradiol, two endogenous oestradiol metabolites, induce DNA fragmentation in Sertoli cells. Andrologia.

[30] Fernandez S.V., Russo I.H., Russo J. (2006). Estradiol and its metabolites 4-hydroxyestradiol and 2-hydroxyestradiol induce mutations in human breast epithelial cells. Int. J. Cancer.

[31] Wang P., Zhu B.T. (2017). Unique effect of 4-hydroxyestradiol and its methylation metabolites on lipid and cholesterol profiles in ovariectomized female rats. Eur. J. Pharmacol..

[32] Zhang Q., Hao X., Sun X., Jia Y.C., Zhu Y.Y., Yang Y.X., Zhu B.T. (2025). 4-Hydroxyestrogen metabolites strongly prevent chemically-induced ferroptotic hepatocyte injury in vitro and in vivo. Eur. J. Pharmacol..

[33] Stanley J.A., Aruldhas M.M., Yuvaraju P.B., Banu S.K., Anbalagan J., Neelamohan R., Annapoorna K., Jayaraman G. (2010). Is gender difference in postnatal thyroid growth associated with specific expression patterns of androgen and estrogen receptors?. Steroids.

[34] Uhlén M., Fagerberg L., Hallström B.M., Lindskog C., Oksvold P., Mardinoglu A., Sivertsson Å., Kampf C., Sjöstedt E., Asplund A. (2015). Tissue-based map of the human proteome. Science.

[35] Karbownik M., Reiter R.J., Burkhardt S., Gitto E., Tan D.X., Lewiñski A. (2001). Melatonin attenuates estradiol-induced oxidative damage to DNA: Relevance for cancer prevention. Exp. Biol. Med..

