# Supplementary material for: 17β-Estradiol and Its Metabolites Induce Oxidative Damage to Membrane Lipids in Primary Porcine Thyroid Follicular Cells—Comparison Between Sexes
Source: Int J Mol Sci. 2025 Dec 6;26(24):11807. doi: 10.3390/ijms262411807 (PMC12732838; doi:10.3390/ijms262411807)
Supplement: Supplementary file 1 [file ijms-26-11807-s001.zip › ijms-3954721-supplementary.pdf]

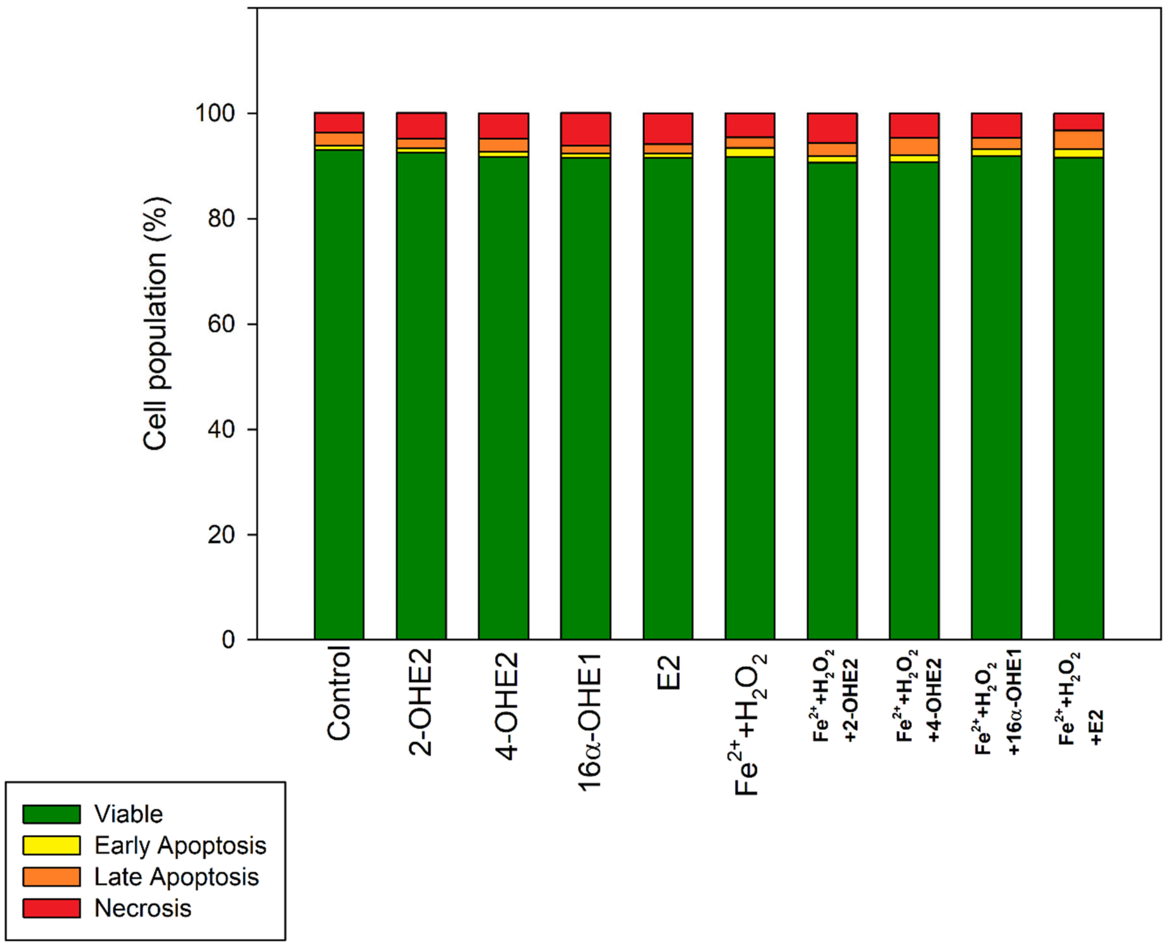

Figure S1. Results of Annexin V/propidium iodide (PI) staining. A cell viability test was conducted using the eBioscience™ Annexin V Apoptosis De-tection Kit (Invitrogen, Waltham, MA, USA; Item No. 88-8005-74) according to the manu-facturer's instructions and analyzed via flow cytometry with a FACSCanto II cytometer (BD FACSCanto II). The data were processed using FACSDiva software 6.1.2 (BD).
